# ‘Maintaining balance in life’—exploring older adults’ long-term engagement in self-managed digital fall prevention exercise

**DOI:** 10.1186/s11556-023-00322-7

**Published:** 2023-07-18

**Authors:** Beatrice Pettersson, Sara Lundell, Lillemor Lundin-Olsson, Marlene Sandlund

**Affiliations:** 1 Department of Community Medicine and Rehabilitation, Physiotherapy, Umeå University, 90187 Umeå, Sweden; 2 Department of Sociology, Umeå University, Umeå, Sweden

**Keywords:** Grounded Theory, Older adults, Aged, Exercise, Fall prevention, eHealth, mHealth, Self-management, Behaviour change, Habit formation

## Abstract

**Background:**

Accidental falls are one of the greatest threats to older adults’ health and well-being. The risk of falling can be significantly reduced with strength and balance interventions. However, there needs to be further knowledge into how older adults can be supported to achieve a maintained exercise behaviour. Therefore, the aim of this study was to explore factors that enabled older adults to maintain their exercise during a 1-year self-managed digital fall prevention exercise intervention.

**Methods:**

This study used a grounded theory methodology. Semi-structured individual interviews were conducted by phone or conference call. Eighteen community-dwelling older adults aged 70 years or more participated. The participants had a self-reported exercise dose of 60 min or more per week during the last three months of participation in a 12-months intervention of self-managed digital fall prevention exercise, the Safe Step randomized controlled trial. Open, axial, and selective coding, along with constant comparative analysis, was used to analyze the data.

**Results:**

The analysis resulted in a theoretical model. We found that the fall prevention exercise habits of adults were developed through three stages: *Acting against threats to one’s own identity, Coordinating strategies to establish a routine, and Forming habits through cues and evaluation*. The main category of *Maintaining balance in life* encases the participants transition through the three stages and reflects balance in both physical aspects and in between activities in daily life. The process of maintaining balance in life and desire to do so were mediated both by intrinsic person-dependent factors and the Safe Step application acting as an external mediator.

**Conclusion:**

This study identified three stages of how older adults developed self-managed fall prevention exercise habits, supported by a digital application. The generated theoretical model can inform future interventions aiming to support long-term engagement in digitally supported and self-managed fall prevention interventions.

**Supplementary Information:**

The online version contains supplementary material available at 10.1186/s11556-023-00322-7.

## Background

The older population is increasing worldwide. This demographic development calls for a shift towards a focus on prevention instead of treatment to support older adults to stay independent and healthy for as long as possible [1]. One of the major threats to older adults’ healthy ageing is accidental falls [2]. One in three community-living adults 65 years and older fall every year, and the frequency increases with age [3]. In 2020, approximately 5% of adults aged 65 and above in Sweden needed care due to fall-related injuries, which are one of the most common causes of death in the older-aged population [4]. The psychological impact of falling and perceived decline in balance, can result in a fear of falling, which can limit participation in everyday activities and physical activity [5], and has a negative impact on experienced quality of life [6].

Efforts can, however, be made to decrease older adults’ risk of falling by providing exercise programs targeting balance and muscle strength [7, 8]. Older adults constitute a diverse and heterogenous group, and it is not feasible to propose a single solution to provide motivation and support for fall prevention exercise programmes. One way to provide continuous support for fall-prevention exercise is through mobile health (mHealth) applications. mHealth applications can support self-management of fall prevention exercise in the person’s own home [9] and has been shown to reduce rate of falls and the number of injurious falls over two years [10]. Although, mHealth applications show promise in providing support for self-management of fall-prevention exercise the research regarding the effectiveness of fall prevention mHealth-interventions are still scarce [11]. Additionally, fall prevention exercise interventions struggle with adoption and adherence [12]. Incorporating behaviour change theories and techniques, such as goal setting and self-monitoring, have shown promise in supporting older adults’ adoption and maintenance of physical activity and exercise [13, 14], although the evidence regarding long-term maintenance is scarce and inconclusive [14]. More research into what factors support continued exercise behaviour is warranted. Therefore, it is important to understand the strategies of older adults who develop and maintain exercise routines.

Habit formation literature provides insight into how a learned behaviour can become habitual. Habits are formed by continuously repeating the same behaviour in a similar context. The context therefore serves as a cue to initiate the behaviour [15]. Through repetition the behaviour becomes progressively autotomized and requires less thought to perform. Habit formation and its determinants have been proposed to be understood as a three-stage process [16]. First, the intention and decision to act is formed (Stage 1). Secondly, the intention is translated into action (Stage 2). Finally, the initiated behaviour needs to be repeated, which requires motivation and self-regulatory strategies (Stage 3a). What distinguishes habit formation is the development of cue-behaviour associations that promotes automaticity (Stage 3b) [15]. Although behaviour change and habit formation theories provide insight into influential factors of exercise behaviour, there is still a significant gap in understanding the process of older adults’ long-term engagement in fall-prevention exercise.

Therefore, the aim of this study was to explore factors that enabled older adults to maintain their exercise during a 1-year self-managed digital fall prevention exercise intervention.

## Method

This qualitative study analysed individual interviews conducted after participants had been exercising with a self-managed digital exercise program, the Safe Step application, for 12 months. Data collection and analysis were conducted simultaneously in an grounded theory approach as described by Charmaz [17]. The Swedish Ethical Review Authority approved the study (Dnr 2021–03894). The study is reported according to the Consolidated criteria for reporting qualitative research (COREQ) [18].

### Study context

The study is based on interviews with a sub-sample of participants from the Safe Step randomized controlled trial (RCT). The RCT protocol and results from the reach and the participant characteristics of the study has been described elsewhere [19, 20]. In short, the RCT was ongoing for 12 months and 1628 participants were randomized in a 1:1 ratio. The primary outcome was establishing fall rate over the 12-month intervention period. The RCT was designed to be fully self-managed, thus all elements of the trial (enrolment, consent, randomization, information about the intervention, information of study procedures, and data collection) were managed through the project website and by e-mail. After responding to a digital baseline questionnaire, the participants were randomly assigned either to exercise with a fully self-managed digital exercise program, i.e., the Safe Step application (v2), and to receive monthly educational videos about healthy ageing and falls prevention, or to receive monthly educational videos only.

The participants in the exercise intervention self-managed their exercise guided by the Safe Step application. At the onset of their use of the program, the participants viewed a short introduction video and were asked to compose their individual exercise program from a repository of predetermined exercise categories. In the application, the participant could view and select the exercises in video format with verbal instructions, performed by an older adult in a home environment. The application also provided videos with examples of when and where to perform the exercises, e.g., different setting outdoors. The participants were informed that their exercise program should contain 10 strength, balance, and mobility exercises that should be strenuous but not too hard to perform. In the exercise videos, participants were encouraged to progress with the exercise when they could complete 2 sets of 10 repetitions with ease, or when the balance exercise was perceived as not challenging enough [19]. When using the application participants were supported to maintain and progress their exercise with behaviour change support such as planning tools, an exercise diary, reminders, and feedback messages from a virtual physiotherapist, as well as additional tips and instructions for integrating exercises into daily activities [21].

### Participants

This study included participants that reported exercising ≥ 60 min per week for the past three months in a questionnaire administered at the end of the 12-month intervention. The initial goal was to include participants who met the study's recommendations of 90 min of exercise/week. As few participants met these recommendations, the limit was changed to 60 min per week as this was still considered a significant amount of exercise with the program. During the last six months of the data collection to the RCT, participants who met the inclusion criteria of exercise dose were sent an invitation via e-mail with accompanying written information about this study and were asked to reply to the e-mail if they were interested. One e-mail reminder was sent. In all, 45 invitations were e-mailed, 19 participants accepted, 1 declined, and 25 did not answer. One interview was excluded because it became obvious during the interview that the inclusion criteria was not met. In total, 18 older adults [16 women, 2 men] aged from 70 to 87 (median 76.5) were included in this study. The participants in this study, as well as the total sample of the RCT were predominantly women, experienced users of internet on their smartphones and tablets, and highly educated [20]. The majority of the participant in this study had experienced a fall during the previous year (78%) and presented varying degrees of physical activity engagement at the beginning of the study (Table 1).

**Table 1:** Participant characteristics at baseline

Variable	Interview study (*N* = 18)	Total RCT (*N* = 1628)
Age, mean ± SD (min–max)	76.1 ± 4.4 (70–94)	75.9 ± 4.4 (70–94)
Women, n (%)	16 (88.9)	1292 (79.4)
Education ≥ 12 years, n (%)	18 (100)	1170 (71.9)
Residency, n (%)		
City	14 (77.8)	1051 (64.5)
Town	2 (11.1)	327 (20.1)
Village or rural area	2 (11.1)	250 (15.4)
Use of Internet or applications on smart technology, n (%)		
Multiple times per day	8 (44.4)	1154 (70.9)
Almost every day, or at least once per week	10 (55.6)	429 (26.4)
At least once per month but not every week, or more seldom	0	45 (2.8)
Falls previous year, n (%)	14 (77.8)	^a^916 (56.3)
Self-rated overall health, n (%)		
Very good or Good	11 (61.1)	863 (53.0)
Fair	6 (33.3)	667 (41.0)
Poor or Very poor	1 (5.6)	98 (6.0)
Prescription medications/day, n (%)		
None	2 (11.1)	235 (14.4)
1–3	12 (66.7)	779 (47.9)
4 or more	4 (22.2)	614 (37.7)
Perceived balance, n (%)		
Very good or Good	4 (22.2)	268 (16.5)
Fair	6 (33.3)	821 (50.4)
Poor or Very poor	8 (44.5)	539 (33.1)
Perceived leg strength, n (%)		
Very good or Good	8 (44.5)	584 (35.9)
Fair	6 (33.3)	703 (43.2)
Poor or Very poor	4 (22.2)	341 (20.9)
Walking aid, n (%)	4 (22.2)	297 (18.2)
Physical activity, n (%)		
Physical daily activities (hours/week)		
< 1	8 (44.5)	626 (38.5)
1–2	2 (11.1)	479 (29.4)
> 2	8 (44.4)	523 (32.1)
Strenuous physical activities (hours/week)		
< 1	13 (72.2)	1214 (74.5)
1–2	4 (22.2)	299 (18.4)
> 2	1 (5.6)	115 (7.1)
TTM, n (%)		
Maintenance	8 (44.4)	832 (51.1)
Action	2 (11.1)	132 (8.1)
Preparation	1 (5.6)	175 (10.8)
Contemplation	4 (22.2)	297 (18.2)
Precontemplation	3 (16.7)	192 (11.8)

^a^ = n = 1626, TTM = Transtheoretical model, Precontemplation = Not engaging in regular exercise and no intention to start in the future, Preparation = Seriously considering to start exercising – has taken some steps toward the objective, Maintenance = Exercising consistently for six months or more

### Data collection

Data was collected through individual interviews [22] between September 2021 and March 2022. The first author, who had previous interviewing experience, conducted all interviews either by telephone (*n* = 15) or video conferencing (*n* = 3) according to the participants’ preferences. The interviews were audio recorded and lasted between 38 and 91 min (mean 61 min). A semi-structured interview guide with open-ended question was used (see Additional file 1). Follow-up questions were focused on providing further insight into the participants’ actions, processes, and experiences. An example of a main question would be: “What strategies did you have to keep the exercise going?” An example of a follow-up question would be: “You told me that reminders were key for keeping the exercise going. Can you tell me why?”. All interviews were transcribed verbatim and analysed using the software MAXQDA 2020. The research team consisted of four physiotherapist researchers with collective expertise in older adults’ health, fall prevention, eHealth, and self-management of physical activity. All had extensive experience in interview studies and qualitative methodologies.

### Analysis

The analysis was performed according to grounded theory through several steps of open, axial, and selective coding in an iterative process of constant comparison of empirical data, codes, and memos [23]. Following Charmaz [17], the analysis focused on capturing the participants’ ‘actions’ and ‘processes’ to achieve long-term engagement in self-managed digital fall prevention exercise. Throughout the analysis, memos were written both in relation to the interview itself after the first reading, as well as to paragraphs, and codes.

Open coding began after the first two interviews. By keeping the codes closely linked to the data, a broad understanding of the data was facilitated. The first author (BP) coded one interview, and all authors met to discuss the codes. Thereafter, all four authors coded one interview independently, and the codes were discussed amongst the authors. BP then coded the rest of the interviews independently.

Due to the iterative analysis process, axial coding was initiated after the third interview had been completed by drawing connections between the initial codes and creating categories [17]. As part of theoretical sampling, interviews in the later stages of data collection were focused on understanding dimensions, properties, and interaction of the formed categories [17]. Through the constant comparison of interviews, memos, codes, and the categories, a core category was formed. All authors met regularly to review and discuss the analysis.

The core category was further refined through selective coding, which sought to find connections between categories and the core category [17]. Relationships became clear, and a theoretical model was generated based on sensitized concepts and was thoroughly discussed amongst the authors. A first draft was written during this stage. Subsequently, a literature review was performed, which showed a significant overlap of the model with habit formation theory [16]. The literature review led to moving one of the subcategories (Coordinating strategies to establish a routine) to its own category. Thus, all stages of a framework of habit formation [15] were represented by the categories. Showing how and why the participants moved through the stages was thought to enrich the model. In the two last interviews no new aspects of the properties of the categories were discovered. At this stage, theoretical saturation was thought to have been reached [17].

## Results

**Fig. 1:**
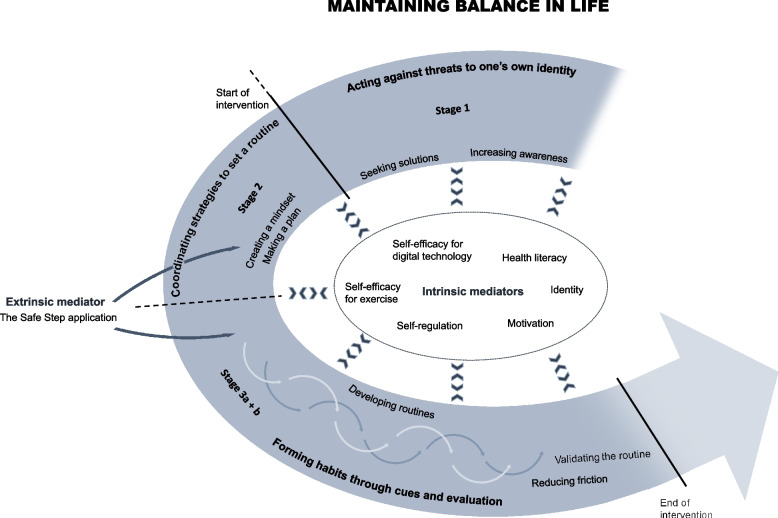
The theoretical model of older adults´ process of forming exercise habits supported by the Safe Step application. The process comprised three different stages reflecting the stages presented in the habit formation framework [15], of which all were permeated by the desire to maintain balance in life

The theoretical model (Fig. 1) that emerged from the data depicts older adults’ experiences of maintaining an exercise routine to prevent falls as a process represented by the core category ‘Maintaining balance in life’. Maintaining balance reflected balance both in physical matters but also an overall balance in life between activities of everyday life as well as a representation of independence. Three categories were identified that represent three stages of establishing exercise habits: ‘Acting against threats to one´s own identity’, ‘Coordinating strategies to establish a routine’, and ‘Forming habits through cues and evaluation’. The stages were informed by intrinsic and extrinsic mediators. The intrinsic mediators were person-dependent and influenced both why and how the process of maintaining balance in life was initiated, but also how the process developed over time. The Safe Step program acted as an external mediator, which facilitated the process of establishing routines and forming habits.

### Acting against threats to one´s own identity

The model’s first stage encompasses the impact on the older adults’ self-perception through gained insights into their risk of falling. Their identity as an independent and capable person was threatened by an affected confidence in performing activities independently and without getting hurt. The category also describes the choice of taking command over the threatening situation by looking for solutions and support to act against the threat of losing balance in life. The intrinsic mediators of health literacy as well as previous experiences and confidence in performing physical activity and managing technology influenced their choice to act, and their decision to enrol in the study.

#### Increasing awareness

The threat to one’s identity was revealed through increasing awareness. This insight could have been gained from either an unexpected turning point or by gradually gaining insight of decline or impairment. The turning point was often an event during which a feeling of vulnerability and shame arose, and which also resulted in a difficulty in trusting one's body in challenging situations. The event could be a fall that resulted in limiting and/or painful injury, or one related to a lingering feeling of concern over what might have happened. The event could also be a gradual decline in bodily function that caused a growing sense of imbalance. The increasing awareness caused growing unease or frustration in performing activities, such as not being able to rise from a chair. This activity in particular was symbolic of the threat against independence and self-perception and was expressed as a catalyst of action.

*“If I can’t stand up, I can’t do anything. I can’t clean, I can’t wash, I can’t lift, I can’t bake. I can’t do anything at home. It’s horrific just to sit. You get depressed to the max. In addition, if I can’t get up, I can’t go anywhere… You get broken. And people will treat you differently, they’ll think your head is as stupid as your legs.” *(Woman, Interview 18)

#### Seeking solutions

By seeking solutions, the older adults acted against the threat to their health and the threat to their views of themselves as independent persons. Their knowledge of the ageing process and potential consequences of an accidental fall mediated seeking both immediate and long-term measures, which was informed by previous experiences of physical activity and technology.

To reduce the perceived risk of falling, immediate measures were taken such as using poles when walking or altogether avoiding what were perceived as high-risk situations for falling. Although longer recovery time was expected after injuries due to older age, states of fear and bodily concern were not accepted as a new normal condition. Maintaining independence was seen as priority, and the sense of being vulnerable was reshaped to decisiveness in seeking solutions.

“I knew that I had to start training to be able to maintain my independence… I simply had to get started. Because they discussed putting me in a home. I would be in a residence. And I don’t want that. If I am placed there, I might as well die. *becomes emotional*” (Interview 17, Woman)

Looking for health advice was a common strategy that could have sprung from previous interest in and the habit of seeking information regarding physical activity, nutrition, and general healthy living. The generated health information, from the internet or counselling through health care, could serve as a mediator to reflect the need of fall prevention measures when gaining insight into deterioration, which was a predisposed factor when receiving information about the Safe Step study. Some older adults had developed a habit of taking up offers of trying different measures to counteract their balance impairments, such as signing up for the study, as they had refused to give up hope of finding a solution to their problems.

### Coordinating strategies to establish a routine

In the model’s second stage, the intention to exercise with the Safe Step program was transformed into action through coordinating strategies by aligning intentions and plans to act. A routine that aimed to create a balance between the new exercise routine and other activities was established. This process was intrinsically mediated by motivation for and previous experiences of physical activity. The Safe Step application acted as an extrinsic mediator, facilitating the onset of the exercise and the making of an exercise plan.

#### Creating a mindset

Creating a mindset with a clear intention of exercise maintenance was important when establishing a routine. This could be done by generating a feeling of commitment to oneself or to the research project. When possessing stronger self-regulatory skills, making a commitment to oneself was enough to initiate the new exercise routine. This mindset was more easily created when it was mediated by a long experience of exercise performance that increased self-confidence in the ability to establish a sustainable routine.

*“Well, I have promised myself that I will do it three times a week and imprinted it in my brain that it must be done. And if it wasn't done in the three days that I had planned from the beginning, it will be three other days.” *(Interview 15, Woman)

#### Making a plan

Making a plan aimed to provide a structure and sustainability for the intended exercise. This plan could be to establish a concrete routine for performing the exercise, or to incorporate it as a part of already existing routines. Adopting a clear structure could be both a strategy based on previous positive experiences of having established routines for exercising, or as a concern of not performing it otherwise. Making a weekly exercise plan could therefore support setting a goal and a commitment to oneself.

*“I think that it’s really important that you’ve decided upon a set time, i.e., after morning coffee. Otherwise it’s so easy that the day just goes on and you do it half-heartedly in the evening because you feel guilty.“ *(Interview 10, Woman)

The clear structure of the Safe Step application further facilitated the making of an exercise plan and supported confidence in performing the exercises, especially for those with less experience in performing physical activity.

*“I thought the description you got in the app were excellent. Because I have a certain tendency to misunderstand things [training instructions]. I thought those were the best explanations I’d seen.” *(Interview 8, Man)

### Forming habits through cues and evaluation

In the third stage of the model, habits were developed by continuously performing the exercise program supported by cues to action, evaluating the exercise routine against one’s expectations, and making the exercise conform to everyday life. Forming habits was a stage of striking and maintaining a balance between exercise and everyday activities. This process was mediated by the older adults’ own self-regulatory capabilities, motivation, and prior experiences, as well as their confidence in performing physical activity and managing digital technology.

#### Developing routines

When initiating the training, a period of establishing preferences in how to manage the program and the exercises followed. The goal was to create seamlessness with other everyday activities in order to produce a long-term commitment.

One way to develop routines was to strictly adhere to the previous established plan, where days and times acted as cues to initiate the exercise. For those with well-established habits for exercising, the new elements from the Safe Step program could be introduced into existing routines, e.g., dog walking or rehabilitation exercises. Push notices from the Safe Step program also mediated training by helping participants remember to exercise on set days and times. The push notices could become imperative for continuing to maintain the exercise, for some, throughout the entire intervention period, especially those with experience exercising and those who found exercise boring.

*“Some say that there’s a natural need to continue to exercise. I don’t have that need! I’m lazybones. I come up with excuses to avoid exercise! I really needed the reminder and the push from the application. “ *(Interview 1, woman)

Participation in the research project could also give rise to a sense of duty. The cue from the application could therefore be perceived as a nudge to act on the commitment. The commitment towards the research project could also be appreciated as it was perceived as easier to maintain the exercise routine with the feeling of contributing to research or of belonging to a social context. This was often because the participant either did not appreciate physical activity in general or wanted motivational support.

In practice, the application could be used in every exercise session, by watching the exercises and performing them simultaneously. This was usually a strategy used at the beginning of the intervention, before learning the exercises. However, over time, this process of starting the application and clicking on each exercise became part of the exercise routine because it required less deliberate thought to just follow instructions, and the company of the older adults on the videos was appreciated.

*“You get some kind of personal relationship [with the older adult performing the exercises], even though I can’t give something back. But, it feels like a nice person exercising with me.” *(Interview I5, Woman)

The exercise routines could change from the entire program being performed in one session to being incorporated into everyday life. Therefore, the support of the application could become less imperative as the exercises were learned by heart. Through repetition, the information regarding the body’s reaction when performing the exercises transformed into bodily knowledge. Bodily sensations, as, for example, stiffness after sitting too long, could therefore signal a need for doing a few repetitions of sit-to-stand. The exercises themselves or the place the exercises were usually performed could act as contextual cues. For example, one could stop to do a few toe lifts while climbing the stairs. Over time, other contextual cues could form, such as continuously doing mobility exercises for the feet while watching TV, or even seeing the phone or the application.

*“I think that it’s good that when you have a lot on your mind and you open the phone, then the app is there and beams. ‘Oh right, I should do my exercises’. Because it's not like I've done it at the same time every day!” *(Interview 16, Woman)

#### Validating the routine

Validating the routine was a parallel process to forming exercise routines and reducing friction that supported developing habits. The perception of the Safe Step application as supportive to the exercise routines was validated by the support it provided for creating the exercise program and its ease of management. However, effects were central to evaluating whether it provided added value in and to life.

The exercise program in itself required continuous validation to attain appropriate strenuousness. This continuous management was mediated by the Safe Step program as the information on how the exercises should feel when performed and when they should become more challenging was perceived as clearly stated, which supported a feeling of competency.

*“It was a bit like, I have never been involved in sports or exercise in my entire life, can I really handle this? And then I discover that it's going really well.”* (Interview 9, Woman)

At the beginning of the intervention, the exercise was driven by the hope of improvements and the purpose of preserving health. After a while, there was an observed improvement in physical function and less fear of falling which validated the exercise program and the routines. Improvement increased confidence in the capability to self-manage the exercise and gave a sense of meaning to the repeated performance. Therefore, the exercise became more joyous to perform and easier to instigate.

*“When I was out with some friends in the woods to pick mushrooms or something, I was not able to join the best parts. The others could walk on a cliff or rock and even descend from them. Earlier I took a step back, but now I dare to join in more often… For me, that’s quality of life.”* (Interview 14, Man)

When experiencing the physical status as unchanged, motivation could also be found in the lack of further bodily deterioration. Counteracting deterioration could feel meaningful due to awareness of or information from health care of the importance of physical activity for healthy ageing, which became even more meaningful due to limitations of other physical activities during the Covid-19 pandemic.

#### Reducing friction

For the exercise routine to be developed into a habit in tune with other parts of life, friction continuously needed to be reduced. This was done by adapting how the exercise program was performed, or by adjusting expectations according to motivation and bodily function. The home-based exercise was in itself a mean to reduce friction because of a preference for exercising alone, or not wanting to or being able to travel to activities. Low exercise thresholds were considered key to maintaining the exercise routine.

*“The technology is available all the time. The development has come to the point where I can decide when I want to do things. Paper training programs are in the past, digital is much better.”* (Interview 3, Woman)

When the exercises were learned, progression could come to a halt as low exercise thresholds were prioritized and comfort was found in knowing the exercises. Therefore, maintaining the life balance and doing some of the exercises was prioritized over following the recommended exercise dose. This could also be the case when the desired improvements were attained and the goal shifted to maintaining advancements. Therefore, the exercises that had helped them to attain the improvements were kept.

*“Yes, I changed some exercises. Probably three times or something like that. I had some exercises I thought worked well with my life, and I stuck to those. “* (Interview 13, Woman)

Due to suspended activities because of the Covid-19 pandemic, home-based exercises were also explored out of necessity, as participants confined themselves to their own homes. Consequently, some perceived it as easier to continue to exercise with the help of the Safe Step application.

## Discussion

We discovered that older adults who maintained a regular fall prevention exercise routine also during the last three months of a one-year intervention did so through factors related to ‘Maintaining balance in life’. The core category reflected the older adults’ striving to maintain their identity as independent persons, which was closely related to the need to keep their physical balance intact. But the balance in life also reflected the need for sustainable exercise routines to be in tune with other parts of their lives and the strategies used by the older adults to achieve and maintain that balance. The process was discovered to reflect constructs of habit formation theory [16], and elements of the theory inspired the model.

The starting point for the older adults’ exercise habits in our study was the experienced need to act against a threat of loss of physical function, which was seen as a prerequisite for independence. The strong driving force to remain autonomous has also been seen in a qualitative study of fall risk perceptions among visually impaired persons, for which the desire for independence was found to be closely linked to a wish to remain the same person they used to be despite ageing [24]. In contrast to our study, this wish can lead to setting up a façade, both to oneself and others, ignoring [25] or rejecting [26] the need for fall prevention measures. Another aspect is that bodily decline and falling can be seen as an unavoidable consequence of ageing [27, 28] leading to a strategy of carefulness to protect oneself from falling [26]. The wish to maintain independent can therefore be seen as a strong mediating factor for older adults’ fall preventive actions.

The participants in our study translated their determination of self-preservation into participation in the Safe Step study. In the past, physical activity and exercise behaviour has been explained by intention, such as in the theory of planned behaviour [29]. However, the intention-behaviour gap has been shown to be hard to overcome when engaging in fall prevention interventions because of changes in inertia [24], or perceived barriers, i.e., practical issues such as transportation, concerns about exercise, or lack of support or interest [30]. Self-regulatory strategies such as goal setting and action planning can be ways to bridge the intention-behaviour gap for physical activity among older adults [31, 32]. Our study shows that when establishing an exercise routine, planning as well as creating a mindset are helpful. Still, too much regulation might cause reluctance [33]. Therefore, presenting a variety of tools and strategies might be preferable for satisfying the feeling of autonomy and supporting older adults’ self-management of exercise. Presenting a variety as well as different combinations of behaviour change techniques might be facilitated through the use of digital technology [34]. Notably, many of the participants in this study were not physically active when enrolling to this study, highlighting the need for interventions to support behaviour change. As found in this study, the Safe Step application helped overcome the intention-behaviour gap because it made it easier to understand how to create an exercise program and perform it. The program also provided support for establishing a weekly exercise goal and plan, which in turn increased self-efficacy. Previous literature has demonstrated the importance of self-efficacy beliefs [35] for adoption of physical activity behaviours in older adults [36].

When forming exercise habits, the participants were cued to action by either the application or their own contextual cues. The exercise habits were formed in consonance with other activities in life by continuously validating and refining the routine. The process of maintaining balance in life was mediated by person-dependent internal mediators and the Safe Step application, which acted as an external mediator. For a behaviour to gradually develop into a habit, the behaviour must be repeated in a stable context to create automation of the execution [15]. When automated, the behaviour is performed efficiently with minimal awareness and exerted control [37]. Although some participants in our study spoke of their exercise routine as an integrated part of life, ‘just like brushing their teeth’, the exercise routines were often deliberated on, validated, and refined. As performance of physical activity and exercise requires conscious thoughts and cannot be fully automated, it can be seen as a complex health behaviour [15]. Physical activity has, therefore, been proposed to be understood as a sequence of simple habitual actions rather than as an unconscious process [38]. A distinction between habitual initiation and habitual performance of exercise has been proposed [39]. When deciding to exercise through unconsciously triggered impulses, the behaviour is called an instigated habit, whereas it is habitually executed when different sub-actions of the exercise are performed without conscious thought. These two initiation strategies can also be seen as reflected in our results as participants spoke about the importance of the push notices as a cue to action, but also, as the exercises were learned they could be interwoven with other activities or elicited by the context and therefore required less thought to perform. The cue or instigation of the habit has been proposed as being more important for exercise frequency than a fully autotomized performance [39].

The results demonstrate that the Safe Step application supported anchoring the exercises to everyday activities, such as rising from a chair, or the reminder from the application, or even the sight of the application itself, which provided a stable context for habits to develop. Suggestions are also given in the application to anchor the exercises to daily events such as performing an exercise while waiting for the kettle to boil, and the exercises are demonstrated in a home environment, which could facilitate a cue-response link. The idea of anchoring fall prevention exercise to embedded activities in daily life has been explored in trials using the Lifestyle integrated Functional Exercise (LiFE) programme [40, 41]. LiFE employs individually delivered strength and balance exercises for older adults and concentrates on using everyday activities as triggers for the onset of training. The program has shown a 31% reduction in the rate of falls for adults aged 70 and above at 12-months follow-up [42]. The LiFE programme has been evaluated in both individuals’ own homes, supported by a trained professional, and in a group setting, followed by two booster calls. No format was found to be superior, and the authors conclude that individual preferences should be considered, although, group sessions constitute a less costly format [43]. In the face of new global challenges of a growing and ageing population, new methods need to be evaluated that can reach and deliver fall preventive exercise to a larger proportion of older adults. Seemingly, as similar results were found regarding the support for developing exercise habits in this completely self-managed and digitally delivered study, future research should further explore the support of applications for creating context associated exercise habits.

### Methods discussion

The individual interview method [22] and the grounded theory approach [17] used in this study made it possible to explore factors related to long-term engagement in self-managed digital fall prevention exercise. The results of this study represent the lived experienced of older adults that have exercised 60 min or more per week with a digital fall prevention programme, more specifically, mainly women, residents of a high-income country, and digitally experienced individuals. However, a strength of the study is that the participants were recruited from all over Sweden. We acknowledge that it would have been preferable to interview more men. We strived for a more even representation of sexes: 10 out of the 45 invited older adults were men. However, the representation of sexes is similar to those of fall prevention trials in general [8].

Due to the large geographical spread, we used telephone interviews or conference calls to conduct the interviews. It cannot be ruled out that other answers could have been presented in face-to-face interviews. The data collection was performed by the first author, who was partly responsible for delivering the intervention. As the project was completely digitalized the researcher had had no previous contact with the participants. However, the participants were aware that the interviewer was a researcher within the research project, which might have influenced their responses. Being familiar with the research study however enabled a better understanding of the participants’ realities and experiences, which in this study facilitated many follow-up questions to be formed based on the participants' narratives. The results of the interviews were also continuously peer-debriefed within the research group to discuss any alterations to the interview guide. The coding of the interviews was mainly carried out by the first author. However, triangulation between members of the research team occurred as each member coded separate interviews and were involved in every step of the analysis process. In this article, the analysis did not include participant checking. It would have been of value to check the results for resonance with the participants' experiences. The composition of the team of authors provided both an insider (BP, LLO, MS) and outsider perspective (SL) in terms of knowledge of fall prevention. Still, we acknowledge that the perspectives and competence of the authors is similar. Another strength is that the generated theoretical model fitted well within the stages and concepts of habit formation theory [16].

The Covid-19 pandemic started during the recruitment period of the Safe Step RCT, and more participants enrolled in the study when the restrictions for adults over 70 years were announced in Sweden [20]. Therefore, the impact of the pandemic was explored in the interviews. The effect on the participants' exercise seems to differ. Some described that exercising with Safe Step replaced other training and made it easier to exercise during the pandemic, some that the pandemic did not affect the decision to participate in the study or their exercise with the application at all.

## Conclusion

The knowledge generated in this study provide an understanding into processes related to long-term engagement with digitally supported fall prevention exercise among community-dwelling older adults. Exercise habits were discovered to be developed in three stages and mediated by intrinsic factors as well as an extrinsic mediator in the form of the Safe Step application. This study demonstrates that a digital application can be supportive in developing fall prevention exercise habits. The theoretical model that was generated can facilitate further exploration and conceptualization of how to support older adults to develop exercise-related cues and fall prevention exercise habits.

## Supplementary Information

**Additional file 1.**

## Data Availability

Data analyzed in the current study are available from the corresponding author on reasonable request.

## Abbreviation

RCT: Randomised controlled trial
