# Supplementary material for: ‘Maintaining balance in life’—exploring older adults’ long-term engagement in self-managed digital fall prevention exercise
Source: Eur Rev Aging Phys Act. 2023 Jul 18;20:12. doi: 10.1186/s11556-023-00322-7 (PMC10354884; doi:10.1186/s11556-023-00322-7)
Supplement: Supplementary file 1 — Additional file 1. [file 11556_2023_322_MOESM1_ESM.pdf]

Recruitment

I am interested to know what made you enroll in the study. Can you tell me a little about that?

What were your expectations of participating in the Safe Step study?

Pandemic

We have had a pandemic for 1.5 years. How have you had to adjust your physical activity during this period?

Has the pandemic facilitated or hindered your participation in the study in any way?

Information videos

Have you learned anything by watching the videos? Tell me more!

Motivation

How has the motivation been for the exercise during this year?

Have you had any strategies for getting the exercise done ?

Can you tell me if anything in the digital program has been a support for maintaining training? If so: What? For what reason?

Effect on health and quality of life

I am interested to know if you feel that participation in the study has affected you or your life in any way.

Person

What kind of relationship have you had with physical activity and exercise throughout your life?

What are your driving forces for being physically active today?

Exercising with Safe Step

What do you think are the factors underlying your ability to exercise regularly during this 12-month period?

Can you tell me how you have perceived the experience of exercising with the Safe Step application this year?

How have you exercised with the Safe Step application?

How did you experience finding and choosing exercises that were just challenging enough for you?

When you go to a physiotherapist, that person is responsible for choosing appropriate exercises and following up on your training. Now that responsibility has been your own. How did you experience it?

Digital literacy

Has participation in the study affected how you use digital technology in any way? How?

Have there been any issues related to the technology?
If yes: What have they been and how have you handled them?

SAFE STEP

INTERVIEW GUIDE

RCT
